# Anti-Fungal Innate Immunity in *C. elegans* Is Enhanced by Evolutionary Diversification of Antimicrobial Peptides

**DOI:** 10.1371/journal.ppat.1000105

**Published:** 2008-07-18

**Authors:** Nathalie Pujol, Olivier Zugasti, Daniel Wong, Carole Couillault, C. Léopold Kurz, Hinrich Schulenburg, Jonathan J. Ewbank

**Affiliations:** 1 Centre d'Immunologie de Marseille-Luminy, Université de la Méditerranée, Case 906, Marseille, France; 2 INSERM, U631, Marseille, France; 3 CNRS, UMR6102, Marseille, France; 4 Department of Animal Evolutionary Ecology, Zoological Institute, University of Tuebingen, Tuebingen, Germany; Massachusetts General Hospital, United States of America

## Abstract

Encounters with pathogens provoke changes in gene transcription that are an integral part of host innate immune responses. In recent years, studies with invertebrate model organisms have given insights into the origin, function, and evolution of innate immunity. Here, we use genome-wide transcriptome analysis to characterize the consequence of natural fungal infection in *Caenorhabditis elegans*. We identify several families of genes encoding putative antimicrobial peptides (AMPs) and proteins that are transcriptionally up-regulated upon infection. Many are located in small genomic clusters. We focus on the *nlp-29* cluster of six AMP genes and show that it enhances pathogen resistance *in vivo*. The same cluster has a different structure in two other *Caenorhabditis* species. A phylogenetic analysis indicates that the evolutionary diversification of this cluster, especially in cases of intra-genomic gene duplications, is driven by natural selection. We further show that upon osmotic stress, two genes of the *nlp-29* cluster are strongly induced. In contrast to fungus-induced *nlp* expression, this response is independent of the p38 MAP kinase cascade. At the same time, both involve the epidermal GATA factor ELT-3. Our results suggest that selective pressure from pathogens influences intra-genomic diversification of AMPs and reveal an unexpected complexity in AMP regulation as part of the invertebrate innate immune response.

## Author Summary

We are interested in how exactly the nematode *Caenorhabditi elegans*, widely used in biological research, defends itself against fungal infection. Like most animals, this worm responds to infection by switching on defense genes. We used DNA chips to measure the levels of all the worm's 20,000 genes and discovered new inducible defense genes. Many of them encode small proteins or peptides that can probably kill microbes. By looking in other nematode species, we saw that these antimicrobial peptide genes are evolving rapidly. This means that they could be important for the worms' survival in their natural environment. We also looked at how some of these genes are regulated and uncovered a sophisticated control mechanism involving a series of proteins called kinases that relay signals within cells. The genes we looked at are active in the worm's skin. Some of the antimicrobial peptide genes that we looked at are also switched on in the skin by high salt, but in this case, the regulation doesn't involve the same cascade of kinases. The responses to both infection and high salt do, however, require the same transcription factor (the protein that actually switches genes on), in this case called a GATA factor.

## Introduction

Two strategies exist for organisms that suffer from predation or infection in their natural environment. They can invest in constitutive defenses that will offer them permanent protection, and they can use inducible defenses that are activated only when they are in danger [1]. In *C. elegans*, the epidermis is at the interface with the environment and is expected to play a key role in defense. It is responsible for the production of the collagen-rich cuticle that surrounds the nematode and provides a permanent physical barrier to pathogens. Some bacteria, such as *Microbacterium nematophilum*, *Xenorhabdus nematophila* or *Yersinia pestis* adhere to the cuticle surface and while not physically penetrating the epidermis cause disease [2]–[4]. On the other hand, nematophagous fungi such as *Drechmeria coniospora* adhere to the cuticle and then infect nematodes directly via the epidermis [5].

In many animal species, infection of barrier epithelia results in the up-regulation of genes encoding antimicrobial peptides (AMPs) and proteins [6]–[9]. Much has been learnt about these inducible defenses through comparative transcriptional profiling. In the case of *C. elegans*, our previous study using microarrays with partial genome coverage showed that *D. coniospora* infection provokes increased AMP gene expression in the epidermis [10].

Many AMPs act by disrupting microbial cell membranes [11]. The efficiency of cell disruption by a single AMP may vary for different pathogens and depends on the exact structure of the microbial surface. Hence, hosts exposed to diverse pathogens may evolve a broader repertoire of AMPs that enhance their defensive potential, if this confers a selective advantage. Such AMP variation is found within the genome of *Drosophila melanogaster*. Of the 20 best-characterised AMPs, eight appear most efficient against fungi, eleven against Gram-negative and one against Gram-positive bacteria [7]. AMP diversity within a single genome may be achieved through gene duplication, a process considered to be one of the most important sources of evolutionary innovation [12]. In this study, we sought to characterize more completely the transcriptional response of *C. elegans* to natural fungal infection. We found that putative AMP genes constitute a major part of that response. We show that one group arose via gene duplication and that these duplicated genes are controlled by a complex regulatory mechanism.

## Results

### The transcriptional response of *C. elegans* to fungal infection

**Figure 1:**
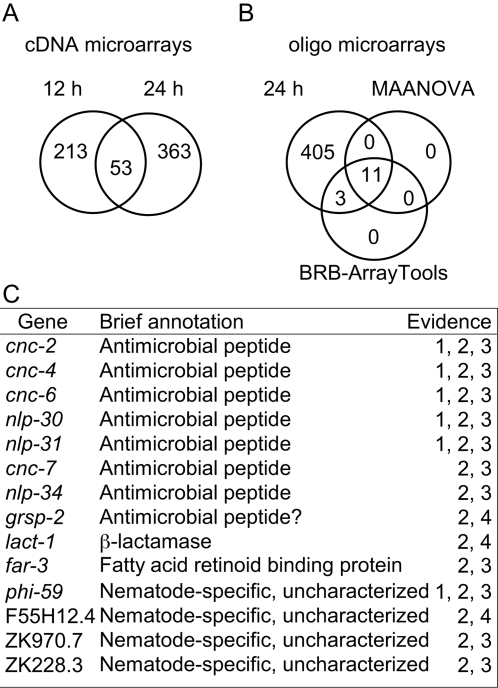
Summary of *C. elegans* genes up-regulated after *D. coniospora* infection. A–B Venn diagrams showing the number of genes identified as robustly up-regulated by *D. coniospora* infection after 12 h and/or 24 h using cDNA (A) and oligo (B) microarrays. C List, with brief annotation, of 14 genes identified as up-regulated at 24 h using statistical methods, (supporting evidence: 1, cDNA microarray; 2, oligo microarray; 3, statistical significance according to both MAANOVA and BRB-Array tool; 4, BRB-Array tools only).

To characterize the response of *C. elegans* to a natural fungal infection, we have analyzed changes in gene expression in worms infected with *D. coniospora*. In a previous study using cDNA nylon microarrays, one striking observation was the increased expression at 12 and 24 h post-infection of multiple genes potentially encoding glycine- and tyrosine-rich antimicrobial peptides (AMPs), members of the NLP (for neuropeptide-like protein) and CNC (Caenacin) families [10]. In our previous report, we only provided data for these *nlp* and *cnc* genes. While they do represent an important component of the response, a large number of other genes are up-regulated at both time points (Figure 1A, Tables S1A & S1B).

The cDNA arrays correspond to fewer than 8,000 of the predicted 20,000 worm genes, so only give a partial coverage of the genome [13]. We therefore carried out an additional analysis using long oligonucleotide whole-genome microarrays [14], comparing the level of gene expression between uninfected controls and worms 24 h after infection with *D. coniospora*. In the top 20 up-regulated genes, ranked by fold-change, (Table S1C), there were 8 *nlp* and *cnc* genes. In addition, there were two previously uncharacterized genes, one that we named *grsp-2* (Glycine-Rich Secreted Protein 2), and the other *fip-1* (Fungus-Induced Protein 1). Inspection of sequences of the next 50 genes led us to annotate 6 other FIPs and 29 FIP-related (FIPR) proteins (see Protocol S1; Figure S1). Based on comparisons to peptides with known antimicrobial activity [15], the *fip*, *fipr* and *grsp* genes could all potentially encode AMPs.

Fold-change measurements are useful when performing exploratory analyses. They can be complemented by methods that evaluate the statistical significance of any observed differences [16]. We used two established statistical-tools, MAANOVA and BRB-ArrayTools (see Materials and Methods) to analyze our data. With the first method, 14 up-regulated and 26 down-regulated genes were found; with the second, 11 and 33, respectively (Figure 1B, Table S1D). Of the 11 up-regulated genes identified with both methods, 7 were *nlp* or *cnc* genes (Figure 1C).

There is data for the expression pattern in uninfected worms for 9 of the 14 genes found to be up-regulated using MAANOVA. As judged by *in situ* hybridization (from the Kohara laboratory) or reporter gene expression, *cnc-2* and ZK228.3 are not expressed at detectable levels, while *far-3* and *cnc-6* are expressed in the intestine. *far-3* is also expressed in epidermis and around the vulva. Most of the genes for which there is data are, however, expressed specifically in the epidermis (Table S1F). Together, these data reinforce the notion that inducible AMP genes expressed in the epidermis are key components of the innate immune response of *C. elegans* to natural fungal infection.

### Evolutionary diversification of AMP genes

**Figure 2:**
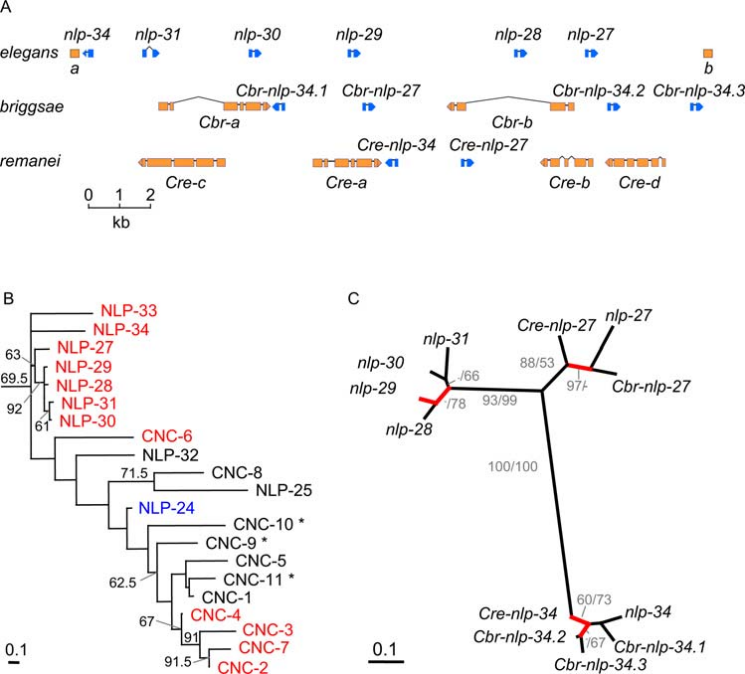
Phylogenetic analysis of *nlp* genes. A The *nlp-29* cluster in *C. elegans* together with syntenic regions from *C. brigssae* (*Cbr*) and *C. remanei* (*Cre*). a, B0213.7 (*str-83*); b, K09D9.9; c, ortholog of B0213.16; d, ortholog of K09D9.10 (*srx-62*); only the 3′ extremities of a and b are shown. B Phylogenetic tree for *C. elegans* CNC and selected NLP proteins, inferred from protein sequences by maximum likelihood (ML). Branches are drawn in proportion to the estimated number of substitutions per site. The results of bootstrapping (200 replicates) are indicated next to the branches. A phylogenetic tree with all the NLP proteins is provided in Figure S2A. The corresponding genes that were found to be up- or down-regulated by *D. coniospora* infection, are represented in red and blue, respectively; those not represented on either array are marked with asterisks. C Unrooted tree for *nlp* genes. Trees were inferred from aligned DNA sequences using ML. Branch-lengths are drawn in proportion to the estimated number of substitutions per site. ML analysis of NLP peptide sequences yielded the same tree with the exception of the exact relationships among *nlp-28* to *nlp-31*. Bootstrap support (500 replicates) is given next to the branches for the peptide (before slashes) and DNA (after slashes) analysis. Branches for which there is support for adaptive sequence evolution are indicated in red; see also Figure S3 and Table S2.

We analyzed the genomic distribution and evolutionary history of *nlp* genes from *C. elegans* and from *C. briggsae* and *C. remanei*, nematodes that belong to a different evolutionary lineage within the *Elegans* group of the genus *Caenorhabditis* [17]. In *C. elegans*, most of the infection-inducible *nlp* genes are found in a 12 kb region on the left arm of chromosome V, that we refer to as “the *nlp-29* cluster”. Through close analysis and re-annotation of the available genomic sequences, we identified syntenic but highly divergent clusters in *C. briggsae* and *C. remanei* (Figure 2A). In *C. elegans*, these genes form an exclusive monophyletic group (Figure S2), separate from the majority of other *nlp* genes that are likely to encode bona fide neuropeptides acting in the nervous system [18]. Within this monophylum, the genes *nlp-27* to *nlp-31* that are immediate neighbours, form a distinct clade (Figures 2A, S2 & S3). These 5 genes, and the adjacent gene *nlp-34*, which together make up the *nlp-29* cluster are all induced by *D. coniospora* infection. We conducted a specific evolutionary analysis on these genes, as well as their respective orthologues from *C. briggsae* (*Cbr*) and *C. remanei* (*Cre*). The inferred phylogeny consistently identified three distinct clades for both the DNA and the protein datasets (Figure 2C & data not shown). In the case of the inferred clades consisting of *nlp-28* to *nlp-31* and also with *Cbr-nlp-34.2* and *Cbr-nlp-34.3*, the constituent genes are adjacent to one another within the respective genomes. This suggests that recent gene duplications occurred within the lineages leading to the different species.

When we tested for adaptive sequence evolution across branches (Protocol S1), we found that non-silent changes are more frequent than silent changes in four branches of the phylogenetic tree (Figure 2C; Table S2). Interestingly, all but one of the identified branches associate with clades of *nlp* genes found adjacent to each other within a single genome (Figure 2C). These results suggest that adaptive sequence evolution shaped the diversification of the *nlp-29* cluster.

### Differential expression of AMP genes in the *nlp-29* cluster in response to infection, injury and osmotic stress

**Figure 3:**
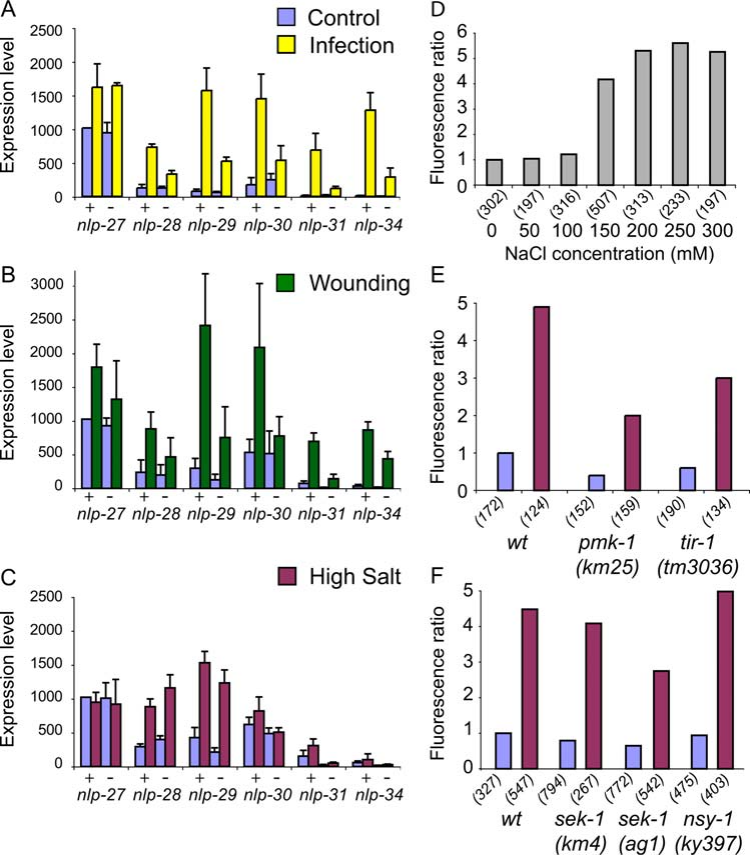
Induction and regulation of nlp gene expression. A–C Quantification of the expression of the genes in the *nlp-29* locus by qRT-PCR after 24 h of infection by *D. coniospora* (A), 2 h after needle wounding (B) and 6 h of osmotic stress in liquid (C). + is wild type; – *pmk-1(km25)*. The columns show the average expression level (arbitrary units, +/− SEM) for at least 3 experiments. The level of *nlp-27* expression in control animals is set at 1024 (see Materials and Methods). Constitutive expression of the *nlp* genes can vary across experiment due to differences in the exact age of the worms or conditions (solid or liquid culture). Within a single experiment, age-matched worms were used. D–F Quantification with the Biosort of the normalized fluorescent ratio (green/red) of worms carrying the integrated *frIs7* transgene that contains p*nlp-29*::GFP and p*col-12*::DsRed reporters [19] after 6 h in liquid culture in the presence of increasing concentrations of NaCl (D) and following a osmotic stress in 300 mM NaCl (E & F; see Materials and Methods). The fluorescent ratio in different backgrounds, *sek-1(ag1), sek-1(km4)*, *nsy-1(ky397)*, *pmk-1(km25)* or *tir-1(tm3036),* for worms after osmotic stress (purple) is compared to control worms (blue). As explained more fully elsewhere [19], due to the nature of the distribution, standard deviations are not an informative parameter and are not shown on this or subsequent figures using the Biosort. The number of worms used in each test is shown in parenthesis. The results shown are representative of at least 3 independent experiments.

Our microarray analyses indicated that all the genes of the *nlp-29* cluster were induced by fungal infection (Figure 2B). We confirmed this using qRT-PCR, and for all the genes except *nlp-27*, observed a statistically significant induction of expression post-infection ranging from 5 to 120-fold (p<0.05; Table S3). On the other hand, and in contrast to the other genes, the level of *nlp-27* in non-infected worms was high (Figure 3A). We have recently shown that *C. elegans* responds to wounding by the up-regulation of *nlp-29* and *nlp-31* expression [19]. Using qRT-PCR, we found that following wounding all the genes in the cluster showed statistically significant increases of expression (p<0.05). It was least pronounced for *nlp-27* (1.6-fold) whereas for the other genes there was at least a 3.5-fold induction. The relative magnitude of these inductions for the different genes was similar to that seen upon infection (Figure 3B; Table S3). Disrupting the cuticle or epidermal cell integrity is therefore sufficient to cause increased expression of all of the genes of the *nlp-29* cluster.

Both infection and injury cause cellular stress. To address the question of whether other stressors provoke AMP expression, we used a reporter strain with an integrated p*nlp-29*::GFP transgene that is strongly induced by *D. coniospora* infection and wounding [19]. We observed no increase in GFP expression when the worms were exposed to a number of stressful situations, including heat shock (1 hour at 37°C, or 10 minutes at 70°C), starvation (for up to 8 hours), paraquat or the heavy metals, cadmium and copper (results not shown). On the other hand, p*nlp-29*::GFP was highly induced by osmotic stress. Thus, exposure of these worms to high concentrations of NaCl (or 100 mM CaCl_2_, MgCl_2_, or MgSO_4_) resulted in an increased level of p*nlp-29*::GFP expression that was dependent on the ionic strength (Figure 3D and results not shown).

We therefore investigated the effect of exposure to high salt on the expression of all the genes of the *nlp-29* locus using qRT-PCR (Figure 3C). We observed a marked increase in the expression of *nlp-28* and *nlp-29* (3.0 and 3.6-fold, respectively; p<0.05), and a smaller, but significant induction of *nlp-31* (2.0-fold; p<0.05). For the other genes of the cluster, *nlp-27*, *nlp-30* and *nlp-34*, no significant changes were observed (p>0.05). Thus, for some of the genes of the *nlp-29* cluster (e.g *nlp-34*), the fold-change in their expression was 2 orders of magnitude higher after infection than it was after osmotic stress, whereas for others (e.g. *nlp-28*) there was an equivalent induction after infection, wounding or exposure to high salt (Table S3).

We obtained results consistent with these qRT-PCR analyses using transgenic strains carrying different reporter constructs. For example, p*nlp-27*::GFP showed a strong constitutive level of fluorescence in the epidermis, while p*nlp-30*::GFP showed an increased level of GFP expression upon infection and wounding, but not osmotic stress (results not shown). These results clearly show that although all genes of the *nlp-29* cluster are induced by natural fungal infection and by wounding, only some are induced by osmotic stress. Thus, the individual AMP genes of the *nlp-29* cluster are subject to differential regulation and respond to distinct combinations of stimuli.

### Differential requirement for the p38 MAPK pathway in the response to infection, injury and osmotic stress

Currently, one of the best characterized innate immunity signaling pathway in *C. elegans* is the p38 MAPK cascade. It is required for resistance to *Pseudomonas aeruginosa* infection [20]–[22]. It involves the MAP3K NSY-1, the MAP2K SEK-1 and the p38 MAPK PMK-1, acting downstream of the conserved adapter protein TIR-1 [23]. This pathway can also regulate the expression of *nlp-29* in the *C. elegans* epidermis. Thus in *nsy-1*, *sek-1*, *pmk-1* or *tir-1*(*tm3036*) mutants, there is essentially no increase in p*nlp-29*::GFP expression after fungal infection or wounding [19]. In clear contrast, *nlp-29* up-regulation triggered by osmotic stress was largely independent of *nsy-1*, *sek-1*, *pmk-1* or *tir-1*, especially when the reduced constitutive expression of the mutants is taken into consideration (Figure 3E & 3F). These results were confirmed by qRT-PCR: the induction by high salt of *nlp-28* and *nlp-29*, the only two genes to be strongly up-regulated by osmotic stress was largely *pmk-1*-independent (Figure 3C; p>0.1 for the difference for either gene between wild-type and mutant). qRT-PCR was also used to demonstrate that the normal response to infection and injury of 5 of the 6 genes in the *nlp-29* cluster required *pmk-1*, the exception being *nlp-27* (Figure 3A & 3B, Table S3). These results indicate that p38 acts to control the response to fungal infection and wounding not only of *nlp-29* but also of 3 other paralogous genes, and *nlp-34*. They also demonstrate that these responses can be genetically separated from the response to high salt.

### Osmotic stress resistant mutants have elevated *nlp-29* expression

We wished to explore further the link between osmotic stress and *nlp-29* expression, to try to understand the physiological role of this regulation. To counter water loss in hypertonic environments, *C. elegans* increases its expression of *gdph-1*. This gene encodes the enzyme glycerol 3-phosphate dehydrogenase that catalyzes the rate-limiting step of glycerol biosynthesis. As a result, intracellular glycerol concentration increases and this has an osmoprotectant effect [24],[25]. Transgenic worms carrying a p*gdph-1*::GFP construct show an enhanced fluorescence upon exposure to increasing concentrations of salt. This reporter gene can thus be used as an *in vivo* sensor of the osmotic stress response [24]. There was no change in p*gdph-1*::GFP expression after *D. coniospora* infection or wounding (results not shown). Thus, these stimuli appear not to trigger an osmotic stress response.

**Figure 4:**
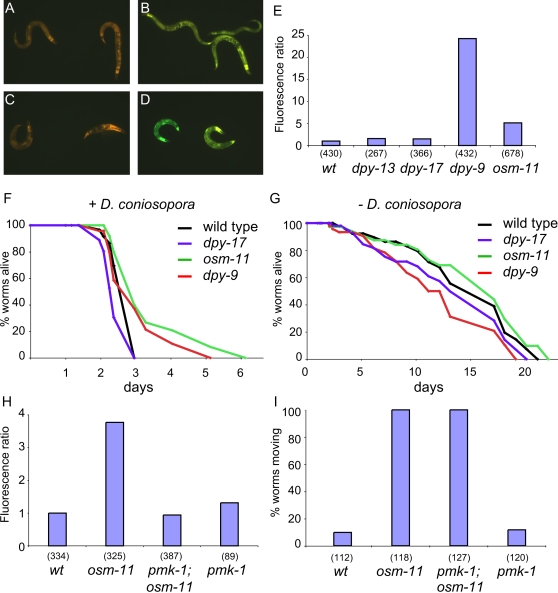
Osmotic stress resistant mutants constitutively express p*nlp-29*::GFP and resist fungal infection. A–D Osmotic resistant mutants such as *osm-11(n1604)* (B) or *dpy-9(e12)* (D) constitutively express p*nlp-29*::GFP while other Dpy mutants including *dpy-13(e184)* (C) behave like wild-type (A) and do not. E Quantification with the Biosort of the normalized fluorescent ratio (green/red) of the integrated *frIs7* transgene in different mutant backgrounds. The number of worms used in each test is shown in parenthesis. F & G *dpy-9* and *osm-11* mutants resist *D. coniospora* infection significantly better than wild-type worms and *dpy-17* mutants (F, p<0.001, one-side log rank test), but do not have a significant different life span in the absence of pathogen (G). Both survival experiments were done on heat killed OP50. H Quantification with the Biosort of the normalized fluorescent ratio (green/red) of worms carrying the integrated *frIs7* transgene in different mutant backgrounds, *osm-11(n1604), pmk-1(km25)* and *osm-11(n1604);pmk-1(km25)*. The number of worms used in each test is shown in parenthesis. The results shown are representative of at least 3 independent experiments. I Acute osmotic stress resistance in wild type worms *osm-11(n1604), pmk-1(km25)* and *osm-11(n1604);pmk-1(km25)* mutants.

Certain mutants, including *dpy-9(e12)* and *osm-11(n1604)* have an elevated level of intracellular glycerol and a higher capacity to resist osmotic stress [24],[25]. We found that these mutants exhibited a high level of p*nlp-29*::GFP expression under normal culture conditions (Figure 4A–E). Dpy mutants are short and fat and manifest alterations of the epidermis and/or cuticle. We tested two other Dpy mutants, *dpy-13(e184)* and *dpy-17(e164)* that have a morphology similar to *dpy-9(e12)* but that are no more resistant to high salt than wild-type worms. They showed normal levels of p*nlp-29*::GFP expression, indicating that an altered morphology is not necessarily associated with elevated AMP expression (Figure 4C & 4E). The two mutants *dpy-9(e12)* and *osm-11(n1604)*, which resist osmotic stress and have a high level of p*nlp-29*::GFP expression, also had a markedly increased resistance to *D. coniospora* infection (Figure 4F, p<0.001), while *dpy-17* mutants were as susceptible as wild type worms. On the other hand, the lifespans of *dpy-9(e12)* and *osm-11(n1604)* mutants are no greater than that of wild type worms in the absence of infection (Figure 4G, p>0.05), suggesting that these mutants exhibit a specific pathogen resistance.

To understand further the link between resistance to osmotic stress and increased *nlp* expression, we first generated a *dpy-9;pmk-1* double mutant. Loss of *pmk-1* function reduced the high constitutive level of p*nlp-29*::GFP expression seen in *dpy-9* mutants (Figure S4). But due to a synthetic interaction, the double mutants retained eggs (Egl phenotype) and were fragile, so that we were unable to carry out more analyses. The *osm-11;pmk-1* double mutant, however, allowed us to assay the contribution of increased *nlp* expression to osmotic resistance. Compared to *osm-11* mutants, these worms had a drastically diminished level of p*nlp-29*::GFP expression, like that of wild-type worms (Figure 4H). On the other hand, the *osm-11;pmk-1* double mutant were still far more resistant to osmotic stress than wild-type worms (Figure 4I) in standard tests of acute osmotic stress resistance (500 mM NaCl) [25]. These results suggest that *nlp-29* is not involved in osmotic protection.

### Overexpression of AMP genes increases resistance to infection but not to osmotic stress

**Figure 5:**
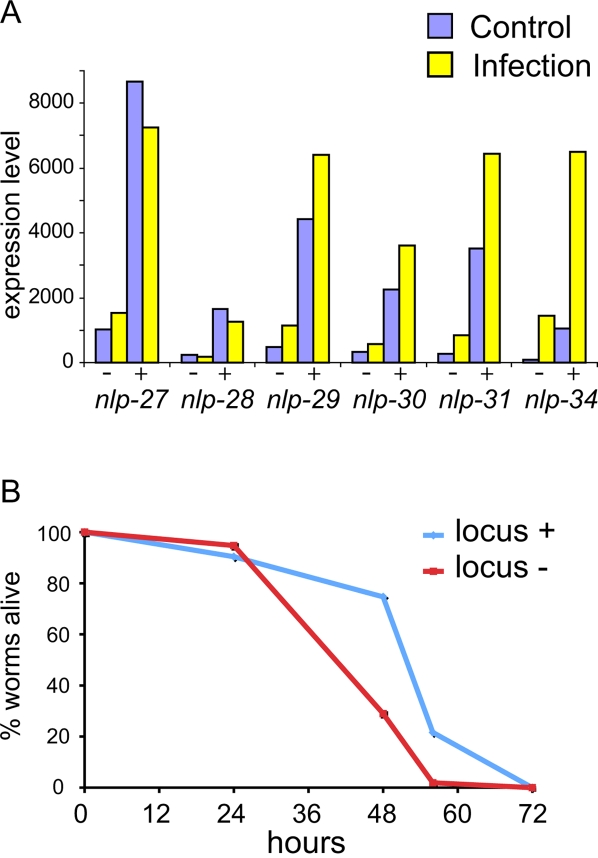
Overexpression of the *nlp-29* locus. A Transgenic worms carrying extra copies of the *nlp-29* locus have elevated levels of gene expression. The level of gene expression for the 6 genes of the *nlp-29* locus was compared between transgenic worms carrying a DNA fragment specifically encompassing the locus (+) and non-transgenic worms (−), either following *D. coniospora* infection (yellow) or not (blue). B Overexpression of the *nlp-29* locus is associated with increased resistance to infection. Transgenic worms carrying the *nlp-29* locus (in light blue) are more resistant to *D. coniospora* infection than their non-transgenic sibling (in red). The difference in survival is highly significant (p<0.001, one-side log rank test).

When we assayed the survival of the null mutant strain *nlp-29(tm1931)*, which cannot make any NLP-29, we saw no marked change in its resistance to *D. coniospora* infection nor its lifespan in the absence of infection (see below). Abrogation of the function of *nlp-31* does not have a significant effect on survival either [10]. In both cases, this could reflect a redundancy in the function of single *nlp* genes in the *nlp-29* cluster, especially given their high level of expression after infection. Therefore, to test whether the genes of the *nlp-29* cluster could contribute directly *in vivo* to the capacity of *C. elegans* to resist infection, we generated transgenic strains carrying supernumerary copies of the entire *nlp-29* cluster. By qRT-PCR we determined that there was an increase in the constitutive and inducible level of gene expression for all 6 genes in the cluster in the transgenic worms (a 3 to 8-fold increase for the different genes, Figure 5A). We then assayed their resistance to *D. coniospora* infection and found a significantly greater survival when compared either to non-transgenic siblings or worms carrying an unrelated transgene (Figures 5B, S5A & S5B; p<0.001). The transgenic worms were, however, as susceptible as wild-type worms to 500 mM NaCl (results not shown). These results indicate that the *nlp* genes can contribute *in vivo* to increased resistance to fungal infection, but probably not to osmotic stress.

### The GATA transcription factor ELT-3 fulfils a generic requirement for *nlp-29* expression

**Figure 6:**
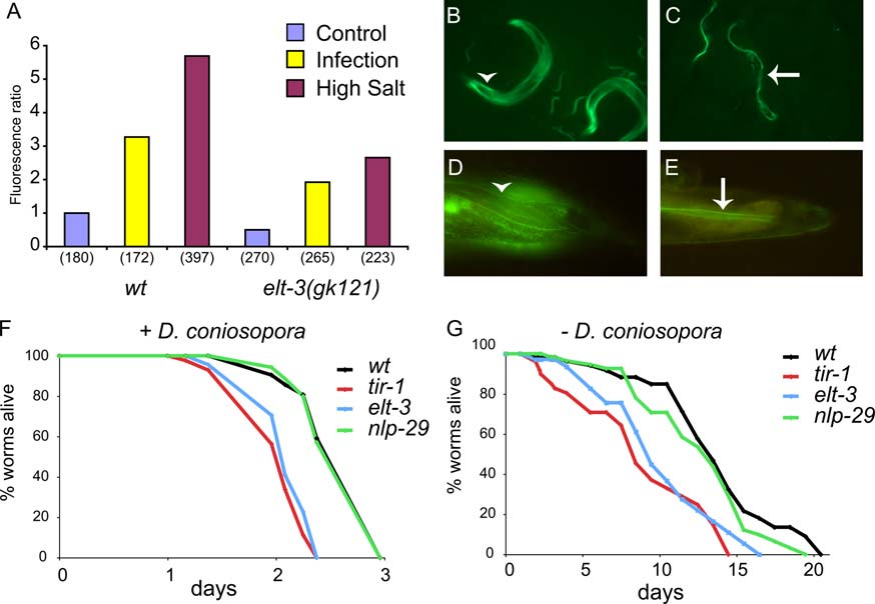
The GATA factor ELT-3 is required for gene induction in the epidermis. A Quantification with the Biosort of the relative fluorescent ratio following infection (yellow) or osmotic stress (300 mM NaCl in liquid) compared to control worms (blue) in *wt* or an *elt-3* background. The number of worms used in each test is shown in parenthesis. Fluorescence images of a p*gpdh-1*::GFP reporter gene in *wt* (B,D) or an *elt-3* (C,E) background after overnight exposure to 200 mM NaCl. The tail region is detailed in D&E. In *wt*, p*gpdh-1*::GFP is induced in both the epidermis (arrowhead) and intestine, as compared to *elt-3* mutant where p*gpdh-1*::GFP is only present in the intestine (arrow). F & G *elt-3 nlp-29 tir-1* mutants are significantly more susceptible to *D. coniospora* infection better than wild-type worms (F, p<0.001, one-side log rank test), but do not have a significant different life span in the absence of pathogen. (G). Both survival experiments were done on heat killed OP50.

Inspection of the upstream sequences of genes of the *nlp-29* cluster revealed the presence of a conserved putative GATA site in the promoter regions of *nlp-28* to *nlp-31* (Figure S6). The GATA factor ELT-2 has been shown to be important for the control of infection-inducible gene expression in the intestine [26]. There are 14 GATA factors encoded in the *C. elegans* genome [27]. We focused on those known to be expressed in the epidermis or seam cells, namely *elt-1*, *3* and *6* and *egl-18* (previously known as *elt-5*) [28]–[30]. RNAi of *egl-18*, *elt-1* and *6* did not have a significant effect (results not shown). We observed, however, that the constitutive expression of p*nlp-29*::GFP and its induction by infection or high salt was reduced upon *elt-3* RNAi. We confirmed this effect using an *elt-3* null mutant allele and found that GFP expression was knocked down by half following either of these treatments, as well as in untreated worms. The level of red fluorescence, from the p*col-12*::DsRed transgene was, on the other hand, essentially the same (+/−15%) in all cases (Figure 6A). To assay for a role of *elt-3* in fungal resistance, we compared the survival of wild-type and mutant worms after *D. coniospora* infection. Unlike the *nlp-29(tm1931)* mutant, which behaved essentially like the wild type, there was a marked reduction in the resistance of the *elt-3* mutants. These mutants, however, had a substantially reduced lifespan in the absence of infection. The same phenotypes were observed for *tir-1(tm3036)* mutants (Figure 6F & 6G). Thus, while being suggestive, we cannot definitively assign a specific role in fungal resistance to *elt-3*.

Exposure to high salt up-regulates expression of the p*gdph-1*::GFP reporter. Unlike p*nlp-29*::GFP that is expressed specifically in the epidermis, it is expressed in both the epidermis and the intestine (Figure 6B & 6D). Interestingly, in the *elt-3* mutant background, an abrogation of the epidermal expression of p*gdph-1*::GFP was seen, while expression was maintained in the intestine (Figure 6C & 6E). This suggests that although *elt-3* is necessary for expression of AMP genes, it acts as a generic transcription factor for inducible genes specifically in the epidermis.

## Discussion

### Transcriptional response of *C. elegans* to fungal infection

In this study, after an unbiased microarray analysis of genes affected by natural fungal infection in the epidermis of *C. elegans*, we focused on putative AMP genes, as they are the most prominent class of up-regulated genes. Synthetic NLP-31 has demonstrated antimicrobial activity *in vitro* against *D. coniospora* [10]. The other infection-induced NLPs and the structurally-related CNCs are therefore candidate AMPs. Our sequence analysis showed that these proteins can be differentiated from most of the predicted NLP proteins. Indeed, it is important to emphasize that NLP-27 to NLP-34 (but not NLP-32) carry the name Neuropeptide-Like Protein only for historical reasons. With regards the many GRSPs, FIPs and FIPRs, while proteins in other species with similar sequences possess antimicrobial activities [15], expression and biochemical analyses are needed to test if the *C. elegans* proteins have such a function.

A very recent study reported changes in host gene expression induced by the nematode-trapping fungus *Monacrosporium haptotylum* [31]. A comprehensive comparison cannot be made as the study with *M. haptotylum* used microarrays with probes to only a few hundred *C. elegans* genes, and of these only 20 are among the list of 800 genes potentially up-regulated by *D. coniospora* (Tables S1B & S1C). Nevertheless, several *nlp* genes, including *nlp-29*, as well as *cnc-4*, were found to be induced by *M. haptotylum* [31]. While these genes are not induced by a number of bacterial pathogens that colonize the nematode intestine [14],[22],[26], another recent report indicates that infection of *C. elegans* by *Leucobacter chromiireducens* may provoke an upregulation of *nlp-29* [32]. This pathogen infects worms via the uterus. A second Gram-positive bacterium, *M. nematophilium*, adheres to the nematode cuticle and causes disease, but does not induce the expression of *nlp-29*, or indeed any of the *nlp* or *cnc* genes [33]. On the other hand, wounding the epidermis also provokes an up-regulation of the expression of genes of the *nlp-29* cluster, albeit via a genetically-distinct signalling pathway [19]. So both the nature of the pathogen and the route of infection likely play roles in determining the host's transcriptional response.

Several of the robustly induced genes are nematode-specific and of unknown function. The predicted ß-lactamase LACT-1, on the other hand, is homologous to prokaryotic proteins that break down antibiotic ß-lactams produced by fungi. One might be tempted to speculate that this protein could act as an intra-cellular sensor for the presence of fungi. Intriguingly, a similar ß-lactamase (LACTB) is encoded in the human genome, but its function is currently unknown [34]. Other induced genes include *far-3* that is induced by *P. aeruginosa* [22], and encodes one of eight related fatty acid- and retinol-binding proteins in *C. elegans* [35]. A class of structurally unrelated fatty acid-binding proteins (lipophorins) plays a role in clotting in arthropods [36], so FAR-3 might contribute directly to tissue repair. Finally, the gene T19B10.2/*phi-59* is also robustly up-regulated upon fungal infection. Abrogation of its function in worms defective for insulin signaling inhibits osmotic stress resistance [37]. So despite the clear dichotomy between the responses to osmotic stress and infection (e.g. the lack of p38-dependence for the former), as discussed below, it is likely that some genes that are induced upon *D. coniospora* infection affect pathogen resistance indirectly, not via antimicrobial effects, but by influencing other aspects of organismal physiology. Infection also provoked the specific down-regulation of many genes (Tables S1A & S1C). A substantial proportion of these genes (including 14 of the 20 most repressed) encode cuticle collagens. This reduction could reflect a general decrease in gene transcription in the epidermis. On the other hand, the expression of many epidermal genes either does not change (e.g. *col-12*) or is increased (e.g. the genes of the *nlp-29* cluster), leaving open the possibility that transcriptional repression could play a specific role in this innate immune response. This must be the subject of future studies.

### Adaptive evolution of innate immunity genes

Parasites and pathogens can represent extremely powerful selective forces because of their ability to evolve rapidly. The resulting diversity of infectious agents favors hosts with a large repertoire of defense responses, including effector molecules with antimicrobial activity. A broad repertoire of AMP genes could evolve via gene duplication [12],[38]. Strong selection on evolution by gene duplication should result in clustered gene families, since gene duplications are usually more frequent across short genomic distances. This has been observed for immune-responsive genes in *Drosophila* [39]. The large majority of clustered gene families in the *C. elegans* genome appears to be associated with a function in the organism's interaction with the environment [40]. Consistent with this hypothesis, several clusters of duplicated genes are induced strongly by *M. nematophilum* infection [33]. Our study identified multiple small clusters of induced immune defense genes. A detailed analysis of the *nlp-29* cluster indicated that it is undergoing rapid evolution. Although a sequence in the *nlp-30* 5′ UTR is found conserved in the 3′ UTR of *Cbr-nlp-27*, the most parsimonious explanation for the difference observed between the 3 *Caenorhabditis* species analyzed is that at the time of divergence of *C. elegans* from the common ancestor of *C. briggsae* and *C. remanei* (estimated at 3.1–12.2 MYR [41]) there were 2 genes at the *nlp* locus. One of them, the ancestral *nlp-27*, gave rise to 5 genes in *C. elegans*, while the other, the ancestral *nlp-34*, gave rise to 3 genes in *C. briggsae*. This is consistent with the presence of single *nlp-27* and *nlp-34* orthologues in the syntenic region of the *C. brenneri* genome (unpublished results). For *C. elegans*, *C. briggsae* and *C. remanei*, the diversification of the *nlp* genes is associated with adaptive sequence evolution, especially in the case of gene family expansions within species lineages (i.e. the four *C. elegans* genes *nlp-28* up to *nlp-31* and the three *C. briggsae nlp-34* genes). The lineage-specific *nlp* expansions could reflect the stochastic nature of gene duplication and subsequent distinct selective pressures on *C. elegans* and *C. briggsae*. In the future, it will be interesting to test whether these differences in *nlp* genes translate into differences in resistance to the pathogens that the two species encounter in the wild.

Since we have shown that introducing supernumerary copies of the *nlp-29* cluster increases survival, the marginal extra cost of carrying an additional *nlp* gene is presumably outweighed by the advantage gained in a hostile environment. Further, we show, rather remarkably, that the *C. elegans*-specific genes *nlp-28* and *nlp-29* are up-regulated not only by infection and wounding but also by osmotic stress. This does not depend on the p38 MAPK pathway, suggesting that successive gene duplications were followed by divergence of regulatory regions, resulting in the co-option of an *nlp* gene by a pre-existing osmotic stress pathway, and the acquisition of a supplementary function that was then retained in *nlp-28* and *nlp-29*. Currently, the genetic control of the osmotic stress response is incompletely characterized. Studies underway in other laboratories to delineate the molecular cascades involved should allow such a hypothesis to be tested in the future.

### Wounding, infection and the osmotic stress response

If wounding and infection were associated with an alteration of the worm's osmotic balance, activating AMP gene expression under conditions of osmotic stress could then be a way to protect *C. elegans* from pathogens without the need to detect the exact nature of the threat. But infection does not affect p*gdph-1*::GFP expression, nor does wounding (unpublished results). Thus, the tissue damage associated with fungal infection or a needle prick does not trigger an osmotic stress response under laboratory culture conditions.

At the same time, there is clearly a link between the response to infection and osmotic stress, exemplified by the up-regulation of certain genes, including *nlp-29*, under both conditions. Further, the genes *dpy-9* and *osm-11*, which affect the osmotic stress response, act as regulators of *nlp-29* expression. While DPY-9 is a cuticular collagen [42], OSM-11 has been proposed to be secreted by the epidermis and to play a sentinel role in monitoring external conditions and mediation of stress responses [25]. If this is the case, under normal conditions, *osm-11* would repress *nlp-29* expression, and upon osmotic stress or infection, *osm-11* activity would decrease, leading to AMP expression.

Importantly, however, the increased expression of p*nlp-29*::GFP seen in either *osm-11* or *dpy-9* mutant is suppressed in *osm-11;pmk-1* or *dpy-9*;*pmk-1* double mutants. By contrast, the *pmk-1* p38 pathway is not involved in the regulation of glycerol levels or acute osmotic stress resistance [25],[43]. Nor is it required for the induction of *nlp-29* under high salt conditions. Thus the induction of *nlp-29* seen in *dpy-9* and *osm-11* worms might arise from a problem of structural integrity and consequent triggering of the p38 pathway in the mutants, independently of the pathway controlling osmotic stress resistance. Therefore, the level of expression of certain AMP genes could be controlled by the balance of negative OSM-11-dependant and positive PMK-1-dependant regulation.

Since the *nlp* genes appear not to contribute *in vivo* to increased resistance to osmotic stress, the physiological reason for their induction by salt remains unclear. And finally, although an increased expression of AMP genes may contribute to the resistance of *dpy-9* and *osm-11* mutants to fungal infection, other factors, most notably high levels of intracellular glycerol, could affect the growth and virulence of *D. coniospora*.

One common facet of the upregulation of *nlp-29* following infection or osmotic stress was the partial dependence upon the GATA factor ELT-3. This transcription factor therefore appears to play a necessary role in the epidermis in the regulation of genes that respond to environmental stimuli. As such, it is different from ELT-2 that has been proposed to have a specific role in the regulation of innate immune genes in the intestine [26]. The response to infection, injury, and osmotic stress is not, however, part of a general stress-response mechanism, since no induction of p*nlp-29*::GFP expression was seen when the worms were exposed to a number of other stressful situations, such as heat shock or starvation. Thus AMP genes appear to contribute directly *in vivo* to the capacity of *C. elegans* specifically to resist infection after epidermal damage.

### Concluding remarks

In the current work we have analyzed the host transcriptional changes associated with natural fungal infection in *C. elegans*. Our findings reinforce the importance of AMP genes in invertebrate innate immunity. The observation that there has been a recent expansion of the AMP-encoding *nlp* genes, together with the evidence for their *in vivo* role and the positive selection of the *nlp*-29 cluster suggest that these genes are important for the survival of *C. elegans*. In conclusion, this study advances significantly our knowledge of host defenses in the nematode and illustrates how new function may arise during evolution through gene duplication and co-option into existing regulatory mechanisms.

## Materials and Methods

### Nematode strains

The mutant strain *sek-1(ag1)* [20] was kindly provided by F. Ausubel, *nsy-1(ky397)* [44] by C. Bargmann, *tir-1(tm3036)* and *nlp-29(tm1931)* by S. Mitani (Japanese National Bioresource Project) and *fer-15(b26ts), sek-1(km4), pmk-1(km25)*, *dpy-9(e12), dpy-13(e184), dpy-17(e164), osm-11(n1604)* and *elt-3(gk121)* by the *Caenorhabditis* Genetics Center (CGC). All strains were maintained on nematode growth media (NGM) and fed with *E. coli* strain OP50, as described [45].

### RNA preparation

Synchronized populations of either *fer-15(b26ts)* or N2 worms were cultivated at 25°C until the mid-L4 stage for cDNA or oligo microarrays, respectively. Worms were then transferred to plates spread with fresh *D. coniospora* spores and harvested after 12 or 24 h, in M9 buffer. In each case, total RNA from 4 independent samples were extracted with Trizol (Invitrogen).

### Identification of differentially regulated genes

We used cDNA microarrays that partially cover the genome (6424 non-redundant cDNA probes). For both 12 h and 24 h fungal infection datasets (4 biological replicates for each time point), genes with background-normalized, photostimulated luminescence (PSL) ratios (infected/control) >1.01 or <0.99 in at least three out of four arrays were initially considered. Normalized data for cDNA arrays can be found in Table S1A. Differentially regulated genes, corresponding to the uppermost 18.75^th^ percentile of each dataset can be found in Table S1B.

We also used oligo microarrays with full genome coverage, containing 23232 features against 20334 unique transcripts. “Per Spot and Per Chip: Intensity Dependent (Lowess) Normalization” in GeneSpring GX version 7.3 (Agilent Technologies) was used to normalize all data (4 biological replicates). Differentially regulated genes based on fold change, corresponding to the uppermost 18.75^th^ percentile of datasets formed using genes with normalized, expression ratios (infected/control) >1.01 or <0.99 in at least ten out of fourteen arrays are shown in Table S1C. The specificity of the probes corresponding to the *nlp* and *cnc* gene is listed in Table S1E. Primary data is deposited at ArrayExpress (E-MEXP-767, E-MEXP-768 and E-MEXP-479).

### Microarray statistical analyses

#### MAANOVA

Various tools as implemented in the software package, J/MAANOVA version 1.0a (http://www.jax.org/staff/churchill/labsite/) were used. Briefly, raw data was normalized using “Joint Lowess intensity-spatial Lowess” transformation. Normalized data was then analyzed with a variant of the “Mixed Effects ANOVA Model”, in which three components of variance were assumed. Two “fixed” components were array specific effects, condition (pathogen or control) and a “random” component was attributed to the different biological replicates used. Within J/MAANOVA, a *F_s_*-test [46] based on the James-Stein estimator [47] was used to identify genes differentially expressed between our two conditions of interest. Robustness of ANOVA data was tested using a permutation test; means were randomly permuted 500 times and test statistics were recalculated for differences between the two conditions. Agreement between ANOVA and permutation test results would indicate the robustness of the ANOVA model. False discovery rate (FDR) control adapted from algorithms discussed by Y. Benjamin [48] and J. Storey [49] was applied to provide 95% confidence.

#### BRB-ArrayTools

A second analysis was performed using tools within BRB-ArrayTools version 3.4.1 developed by the Biometric Research Branch of the US National Cancer Institute (http://linus.nci.nih.gov/BRB-ArrayTools.html). Lowess intensity dependent normalization was initially used to adjust for differences in labeling intensities of the Cy3 and Cy5 dyes. The adjusting factor varied over intensity levels [50]. Subsequently using “Class Comparison” with dye-swapped experiments being averaged, we identified genes that were differentially expressed among two classes, infected and control, by using a multivariate permutation test. We used this test with 90% confidence so that the false discovery rate was less than 10%. The false discovery rate is the proportion of the list of genes claimed to be differentially expressed that are false positives. The test statistics used were random variance t-statistics for each gene [51]. Although t-statistics were used, the multivariate permutation test is non-parametric and does not require an assumption of Gaussian distributions.

### Phylogenetic analyses

The general phylogenetic position of the antimicrobial *nlp* and *cnc* genes was reconstructed in relation to the remaining *nlp* genes from *C. elegans*, using peptide sequences. For the *nlp-29* clade including the *C. elegans* genes *nlp-27* to *nlp-31* and *nlp-34*, phylogenetic relationships with their 4 orthologues from *C. briggsae* and 2 from *C. remanei* were inferred from both peptide and DNA sequences. Sequences were obtained from Wormbase (www.wormbase.org) Syntenic regions were identified for *C. remanei* and *C. briggsae*. They were re-annotated manually using Blast and the new gene predictions submitted to Wormbase. All alignments were generated with the help of CLUSTALW [52]. Phylogenetic analysis was based on the maximum likelihood (ML) approach. The optimal substitution model was identified with the Akaike information criterion and the program Prottest 1.3 for protein and the program Modeltest 3.7 for DNA sequences [53]-[55]. It was then employed for ML tree reconstruction using the program PHYML [56],[57] for protein and the program PAUP* 4.0b10 for DNA sequences [58]. The robustness of the inferred topology was assessed with the help of non-parametric bootstrapping [59], based on 500 replicate data sets.

The presence of adaptive Darwinian evolution (i.e. mutations that lead to amino acid changes are selectively favored) was assessed for the *nlp-29* clade by analysis of the non-synonymous versus synonymous substitution rate ratio (*d_N_/d_S_*) across the different branches of the phylogenetic tree. We focused on the peptide regions without the signal sequence, where selection for diversifying functions is expected. Based on the program PAML 3.15 [60], two different approaches were employed [61]. On the one hand, we inferred *d_N_/d_S_* ratios for each individual branch using the free-ratio model. Ratios larger than one indicate adaptive sequence evolution. Their significance was tested by non-parametric bootstrapping using 100 replicate data sets. On the other hand, we compared the likelihood of test trees, in which one branch was allowed to vary in *d_N_/d_S_* ratio (2-ratio model), to the likelihood of the null model tree, in which all branches had identical *d_N_/d_S_* ratios (1-ratio model). If the varying *d_N_/d_S_* ratio was larger than one and if the difference between test and null model tree was significant according to a likelihood ratio test (LRT) [60],[62], then this was taken as an indication for the presence of adaptive sequence evolution.

### Reporter gene constructs and generation of transgenic lines

The *frIs7* transgenic strain containing the p*nlp-29*::GFP and p*col-12*::DsRed reporters is described elsewhere [19]. p*nlp-27::*GFP, p*nlp-30::*GFP and p*nlp-31::*GFP [10],[18] were injected at 20 µg/ml into N2 with the transformation marker p*col-12*::DsRed (80 µg/ml). At least three independent lines for each p*nlp::*GFP reporter were characterized. The IG615 (*frEx217*) transgenic strain for overexpression of the *nlp* locus was produced by injecting the entire *nlp* locus into wild type N2 worms with the co-injection marker pB*unc-53*::GFP [63]. The entire locus was included in a 11 kb *Spe*I-*Cla*I fragment from the cosmid B0213 that was purified after migration on 0.4% agarose gel into a buffer-filled trough cut in the gel [64].

### Infection, wounding and osmotic stress

Infections with a freshly harvested solution of *D. coniospora* spores were done as described [65]; worms were analyzed after 24 h at 25°C. Worms were pricked in the tail region using a microinjection needle under a dissecting microscope and analyzed 6 h later for GFP induction or 2 hours later for qRT-PCR. Osmotic stress was done in liquid by incubating young adult worms in 300 mM NaCl for 6 h, or on NGM plates containing 300 mM NaCl after RNAi treatment with analysis 24 h later.

### Analyses with the Biosort worm sorter

Upregulation of p*nlp-29*::GFP reporter gene levels were quantified with the COPAS Biosort (Union Biometrica). Generally 100 to 2000 synchronized worms were analyzed for size (TOF), extension (EXT), green (GFP) and red (RFP) fluorescence (see [19], for further details). The fluorescence ratio (Green/Red) was then calculated to normalize the GFP for variations in size and health of individual worms. Mean values for FR were calculated and the values for the different samples within a single experiment normalized so that the control (*wt;frIs7*) worms had a fluorescence ratio of 1. As discussed more extensively elsewhere, [19] direct numerical comparisons can be made between age-matched populations in single experiments, and qualitative comparisons can be made between experiments performed on different days. The results shown are representative of at least 3 independent experiments.

### qRT PCR

2.5 µg of total mRNA from infected and non-infected worms were used for a reverse transcription using a standard protocol. Primers were designed to detect specific transcripts (see Protocol S1). Using 1/50 of cDNA in 12.5 µl of SYBRgreen mix (Applied Biosystem) and 0.3 µM of primers, qRT PCR were performed on a Gene Amp 5700 Sequence detector. Results were normalized to *act-1*, and then relative expression calculated using 2^((A+10)−x)^, A being the normalized cycle number for *nlp-27* in the non-infected sample and x the value of interest. Control and experimental conditions were tested in the same run. Means and standard deviations were calculated from a minimum of 3 independent experiments. Statistical analyses used the paired bilateral Student's test within Excel (Microsoft software) (Table S3).

### Infection and osmotic assays

50–70 worms at the L4 stage were infected at 25°C with *D. coniospora* and the surviving worms were counted every day as described elsewhere [65] except that the NGM plates were seeded with heat killed OP50. Killing assays were conducted at 25°C. Statistical analyses used one-sided log rank test within Prism (Graphpad software). The resistance to acute osmotic stress was assayed after 10 min on NGM plate containing 500 mM NaCl with young adult worms as described [25]

## Supporting Information

Protocol S1 Supporting Materials and Methods
(0.03 MB DOC)

Figure S1 New classes of putative anti-microbial peptides and proteins. Raw output from CLUSTALW multiple sequence alignments for GRSP, FIP and FIPR protein sequences. The genes identified as being strongly up-regulated by *D. coniospora* are highlighted in yellow.
(0.05 MB DOC)

Figure S2 Phylogenetic tree for the *C. elegans* NLP and CNC proteins, inferred with maximum likelihood from protein sequences. Branches are drawn in proportion to the estimated number of substitutions per site. The results of bootstrapping (200 replicates) are indicated next to the branches. Only values of at least 50 are shown.
(1.44 MB EPS)

Figure S3 Tree topology for the *nlp* genes with branch labels as tested for the presence of adaptive sequence evolution (see Table S2).
(0.95 MB EPS)

Figure S4 The high expression of *nlp-29* in *dpy-9* mutants is almost entirely dependant on *pmk-1*. Fluorescent pictures (A) and quantification with the Biosort of the normalized fluorescent ratio (green/red) (B) of worms carrying the integrated *frIs7* transgene in different mutant backgrounds, *dpy-9(e12), pmk-1(km25)* and *dpy-9(e12);pmk-1(km25)*. The number of worms used in each quantification is shown in parenthesis.
(1.84 MB EPS)

Figure S5 The presence of the cosmid containing the *nlp-29* locus increases resistance to infection. (A) Worms were infected by *D. coniospora*, then transferred to OP50 seeded NGM plates containing the anti fungal agent nystatin (12.5 µg/ml) after 3 or 12 h. Transgenic worms carrying the cosmid B0213 (in black) or their non-transgenic sibling (in red) were scored as live or dead over 6 days. In both cases, the difference in survival is highly significant (p<0.001, one-side log rank test). The IG368 (*frEx75*) transgenic strain contains the whole cosmid B0213 including the *nlp* locus and the co-injection marker *sur-5*::GFP [66]. (B) The resistance of strain CX6760 (*wt; kyEx749*[*F13B10.1;ofm-1::*GFP]) [67] is indistinguishable from that of wild-type worms under standard conditions [68].
(0.66 MB EPS)

Figure S6 Alignment of the promoter sequences of the genes from the *nlp-29* locus. Raw output from CLUSTALW multiple alignments for the proximal 500 bp 5′ sequences for the 6 genes of the *nlp-29* cluster. Putative minimal GATA sites are highlighted in yellow; one is shared between 3 *nlp* genes.
(0.06 MB DOC)

Table S1 Expression patterns of 14 selected genes.
(0.82 MB XLS)

Table S2 Results of the analysis of adaptive sequence evolution for individual branches of the *nlp* tree.
(0.06 MB DOC)

Table S3 qRT-PCR.
(0.04 MB XLS)
