# Supplementary material for: Anti-Fungal Innate Immunity in C. elegans Is Enhanced by Evolutionary Diversification of Antimicrobial Peptides
Source: PLoS Pathog. 2008 Jul 18;4(7):e1000105. doi: 10.1371/journal.ppat.1000105 (PMC2453101; doi:10.1371/journal.ppat.1000105)
Supplement: Protocol S1 — Supporting Materials and Methods (0.03 MB DOC) [file ppat.1000105.s001.doc]

# SUPPLEMENTARY MATERIALS & METHODS

**Microarray hybridization and scanning**

Hybridization was done using an adapted version of a protocol from the Genomics Core Laboratory at the J. David Gladstone Institutes, California, USA. 5 µg of RNA was converted to double-stranded cDNA with superscript II (Invitrogen) using custom designed (dT)_24_-V primer (Sigma) and aminoallyl-dUTP (Sigma) nucleotide analogs. The cDNA was then cleaned using Qiagen PCR purification kit. Then labeled cDNA probes were prepared via coupling to Cy3 or Cy5 mono-reactive dye packs (Amersham). After removal of unincorporated dyes with a Qiagen PCR purification kit, two differentially labeled probes were combined in a hybridization buffer containing 5X SSC, 0.2% SDS, 7mM Tris-Cl, 0.2mg/mL yeast t-RNA (Invitrogen), 0.2mg/mL poly (A) DNA (Sigma). 55 µL of this cocktail was used on each chip and incubated at 45°C for 16 h in a water-bath. The arrays were the processed according to recommended protocols for UltraGAPS™ arrays. Microarrays were scanned using a SCANARRAY 4000XL (Perkin Elmer) and image analysis was performed using QUANTARRAY version 2.1 (Perkin Elmer). Spots of the array with obvious blemishes were manually flagged and excluded from subsequent analyses. Global array quality was kept consistent with normalization coefficients for the fluorochrome channels controlled at <2, as visualized with ArrayPlot version 3.0 [1].

**Identification of FIP and FIP‑related (FIPR) family members**

Inspection of the predicted sequences of putative proteins in the top 21-70 up-regulated genes, ranked by fold-change, led us to annotate 5 FIPs (FIP‑2 to 6) with at least 3 of the 4 following criteria: (i) less than 100 amino acids, (ii) predicted signal peptide, (iii) simple amino acid structure (iv) having a homologue in the immediate genomic proximity, or similar in sequence to more than one structurally-related protein encoded by clustered genes. We identified 29 FIP‑related (FIPR) proteins. A 7^th^ FIP (FIP‑7) is 95 amino acids long, but has the distinction possessing neither a signal sequence, nor homologues even in the closely related species *C. briggsae* and *C. remanei* (Supplementary Figure 1).

**Primers**

Primers used for qRT PCR (with amplicon size) were designed to detect specific transcript:

*nlp-27* (149bp), JEP965 CGGTGGAATGCCATATGGTG and JEP966 ATCGAATTTACTTTCCCCATCC;

*nlp-28* (124 bp), JEP967 TATGGAAGAGGTTATGGTGG and JEP968 GCTAATTTGTCTACTTTCCCC;

*nlp-29* (147 bp); JEP952 TATGGAAGAGGATATGGAGGATATG and JEP848 TCCATGTATTTACTTTCCCCATCC;

*nlp-30* (187 bp), JEP948 TATGGAAGAGGATATGGTGGATAC and JEP949 CTACTTTCCCCATCCGTATCC;

*nlp-31* (160 bp), JEP950 GGTGGATATGGAAGAGGTTATGGAG and JEP953 GTCTATGCTTTTACTTTCCCC;

*nlp-34* (103 bp), JEP969 ATATGGATACCGCCCGTACG and JEP970 CTATTTTCCCCATCCGTATCC

*act-1* (134 bp), JEP538 CCATCATGAAGTGCGACATTG and JEP539 CATGGTTGATGGGGCAAGAG.

**Reference**

1. Marc P, Jacq C (2002) Arrayplot for visualization and normalization of cDNA microarray data. Bioinformatics 18: 888-889.
