# Supplementary material for: Anti-Fungal Innate Immunity in C. elegans Is Enhanced by Evolutionary Diversification of Antimicrobial Peptides
Source: PLoS Pathog. 2008 Jul 18;4(7):e1000105. doi: 10.1371/journal.ppat.1000105 (PMC2453101; doi:10.1371/journal.ppat.1000105)
Supplement: Figure S1 — New classes of putative anti-microbial peptides and proteins. Raw output from CLUSTALW multiple sequence alignments for GRSP, FIP and FIPR protein sequences. The genes identified as being strongly up-regulated by D. coniospora are highlighted in yellow. (0.05 MB DOC) [file ppat.1000105.s002.doc]

Figure S1

C34D4.11 GRSP-3 MKKLALFVLFLTFFETYAQWG-----------------WGP----------------PSP 27

Y43F8C.20 GRSP-1 MQKLTILLLLTILGLATCQWGP--------------GGWGGG---------------PGR 31

F07C4.7 GRSP-4 MYKLTIFLLVTLLGLTVGQWGPGFGG----------PGFGGPGSGG-----------PGP 39

T28C6.1 GRSP-2 -MRLTLLLLAVVLAYVAAQGDGLFGGSDAGSGIGSSGGWGGSDASAGASAGGTGGGRGGG 59

:*::::* : . * . :* .

C34D4.11 GRSP-3 WGFRPSPFFGGRSGGWGRPG-WGG-----GGPGWGR-GGG--GSGWGGGRGGG------- 71

Y43F8C.20 GRSP-1 WGG----WGGNRWGGGGGPGGWGNN----GGGGWGR-GGGRGGGDWGGNNGGGGN----- 77

F07C4.7 GRSP-4 RGPGEWNNGGGR-GGFGGNNGWNN-----GGGGFNG-NGGFNGGGRGGGRGGNGG----- 87

T28C6.1 GRSP-2 RGGSGGGRGGGSGGGRGGSGGAGAGGSGSGSGGWGGQDGGSSAGGWGGSQGGSQGGSSGG 119

* *. ** * . . *. *:. .** ... **..**.

C34D4.11 GRSP-3 WGNN-----GGG-GNWGGNGGGGNGGGGRG-------GGGG--GGGGR------------ 104

Y43F8C.20 GRSP-1 WGGN-----GGGRGDWGGNGGGGRGGGGRGDWGGNNNGGGGNWGGGGNNDGG-------- 124

F07C4.7 GRSP-4 FGDN-----GGL---RGGNGGGRGGNGGFDD-----NGGGRGGNGGGRG----------- 123

T28C6.1 GRSP-2 WGGSSRSDSGSGQGGWGGQQGGNSNAGGWGGSQGGQNGGGGRGGSGGQGGWGGSQDGGSQ 179

:*.. *. **: ** . ** . *** ..**.

C34D4.11 GRSP-3 -------GGGGGGR--GGGGGGGGGRG---------------------GGG--GGRGGGG 132

Y43F8C.20 GRSP-1 --WGGNNGGGGGGR--GGGGRGGDGRGPPG----------------SNGGGDWGGNGGGG 164

F07C4.7 GRSP-4 -------GTGGFGD--NGGGRGGNGGG---------------------RGGNGGGNNGGG 153

T28C6.1 GRSP-2 GGWGGQNGGGQGGNQGGGGGRGGNQGGEQGGWGGQGGSQGGSQGGSQGGWGNQGGQQGGG 239

* * * .*** **. * * **. ***

C34D4.11 GRSP-3 RGRG-------------------------------------- 136

Y43F8C.20 GRSP-1 RGGGGGRGGGGGGGAGERIAEGVLGALLG------------- 193

F07C4.7 GRSP-4 RGGNG--GGRPQISAGERITHNVLGALLG------------- 180

T28C6.1 GRSP-2 RGGQQGPGGWGGGGRGGGWGGWGRGSRWGWGRPSWGGWGRGW 281

**

F23H12.8 FIPR-1 ------------------------------------------------------------

F23H12.9 FIPR-2 ------------------------------------------------------------

F23D12.6 FIPR-3 ------------------------------------------------------------

C12D8.14 FIPR-4 ------------------------------------------------------------

C12D8.18 FIPR-5 ------------------------------------------------------------

C12D8.17 FIPR-6 ------------------------------------------------------------

C12D8.16 FIPR-7 ------------------------------------------------------------

C12D8.6 FIPR-8 MDIIMRLLSASECFETNLELILLLFLFVFYPDFLEKVYHKNKNDVVKDMDICGINEELDN 60

C12D8.19 FIPR-9 ------------------------------------------------------------

C50H2.12 FIPR-10 ------------------------------------------------------------

C50H2.10 FIPR-11 ------------------------------------------------------------

T07C12.13 FIPR-12 ------------------------------------------------------------

F57A8.8 FIPR-13 ------------------------------------------------------------

F22B7.4 FIP-1 ------------------------------------------------------------

T27E4.4 FIP-2 ------------------------------------------------------------

F23H12.8 FIPR-1 ---------------------------------------------MNAKLLILVSLLV-- 13

F23H12.9 FIPR-2 ---------------------------------------------MNAKLLILVSLLV-- 13

F23D12.6 FIPR-3 -----MLKVDWYK----SEFIFTVG-----KNFHILLQLN---FIMNAKFLLCVLALV-- 41

C12D8.14 FIPR-4 ---------------------------------------------MNAKFLLCVLAIA-- 13

C12D8.18 FIPR-5 -----MSEQTGKRVKMYTHFGYKSGN--FWENKQGPSHSSITQFIMNSKFLLCVLAIA-- 51

C12D8.17 FIPR-6 ---------------------------------------------MNSKFLLCVLAIA-- 13

C12D8.16 FIPR-7 ---------------------------------------------MNSKFLLCVLVIA-- 13

C12D8.6 FIPR-8 IAVADMTEQQAHRLLRENQLAWKKEGTKIRRKQRRALKASKRRHEMGMVCLLCVLAIA-- 118

C12D8.19 FIPR-9 ---------------------------------------------MNAKFLLCVLVIA-- 13

C50H2.12 FIPR-10 ---------------------------------------------MNAKFLLCVLALA-- 13

C50H2.10 FIPR-11 ---------------------------------------------MNAKFLLCVLALA-- 13

T07C12.13 FIPR-12 ---------------------------------------------MNAKFLLCVLAFA-- 13

F57A8.8 FIPR-13 ---------------------------------------------MNAKLLILIFTIL-- 13

F22B7.4 FIP-1 ---------------------------------------------MSQIFQILVIFAI-- 13

T27E4.4 FIP-2 ---------------------------------------------MKAILFLVLLVAISW 15

* : :

F23H12.8 FIPR-1 -ICAFVA-----ETDAQYYG-----YGYGGYGYPSSYYGYG-----------------SY 45

F23H12.9 FIPR-2 -ICAFIT-----ETDAQYYG-----YGYG---YPSSYYGYGY---------------GNY 44

F23D12.6 FIPR-3 -MCSIVQ-----DASAQYYG-----YASS--YYPSYYGGYGY---------------GAY 73

C12D8.14 FIPR-4 -MCSIVQ-----EASAQYYG-----YASS--YYPSYYGGYGY---------------GAN 45

C12D8.18 FIPR-5 -MCSIVR-----EASAQYYG-----YASS--YYPSYYGGYGY---------------GAY 83

C12D8.17 FIPR-6 -MCSIVQ-----EASAQYYG-----YASS--YYPSYYGGYGY---------------GAY 45

C12D8.16 FIPR-7 -MCSIVQ-----EASAQYYG-----YASS--YYPSYYGGYGY---------------GAY 45

C12D8.6 FIPR-8 -MCSIVQ-----EASAQYYG-----YASS--YYPSYYGGYGY---------------GAY 150

C12D8.19 FIPR-9 -MCSIVQ-----EASAQYYG-----YVSS--YYPSYYAGYGY---------------GAY 45

C50H2.12 FIPR-10 -ICSIVQ-----ETSAQYYG-----YSS---YYPSYYGGYGL---------------GAY 44

C50H2.10 FIPR-11 -MCSIVQ-----ETSAQYYG-----YSS---YYPSYYGGYGP---------------GAY 44

T07C12.13 FIPR-12 -MCSIVQ-----ETSAQYYG-----YSS---YYPTYYGGYGP---------------GAY 44

F57A8.8 FIPR-13 -TITSGQYYGGYPTASGYYGN--GVYGGG--YGNVGYGGYGYP--------------GQY 54

F22B7.4 FIP-1 --LSALQ------VNGFLFP----TYSSG-----YDYDCYGYG----------NNG---- 42

T27E4.4 FIP-2 TLLDVVD------SKGVPAKRVKRQYYGG-----YGYGGYGYGGCNCPTYAPCTHGPGGY 64

. * * **

F23H12.8 FIPR-1 YGY-GYPYYGGY-GYYGKREAGFGPSQQQNNQ 75

F23H12.9 FIPR-2 YG--SYPYYGGY-GYYGKREAGFGPSQQ-NNQ 72

F23D12.6 FIPR-3 GAYGGYGLGSAYAGYYGKREAGFGPVQN---- 101

C12D8.14 FIPR-4 GAYGGYGLGSAYAGYYGKREAGFGPIQN---- 73

C12D8.18 FIPR-5 GAYGGYGFGSAYAGYYGKREAGFGPTQN---- 111

C12D8.17 FIPR-6 RAYGGYGLGSAYAGYYGKREAGFGPTQN---- 73

C12D8.16 FIPR-7 GAYGGYGLGSAYAGYYGKREAGFGLTQN---- 73

C12D8.6 FIPR-8 GAYGGYGLGSAYAGYYGKREAGFGPTQN---- 178

C12D8.19 FIPR-9 GTYGGYGLGSAYAGYYGKREAGFGPTQN---- 73

C50H2.12 FIPR-10 NGYYGGAYGYGGYGYYGKREAGFGPSQQAN-- 74

C50H2.10 FIPR-11 NGYNGGAYGYGGYGYYGKREAGFGPSQQAN-- 74

T07C12.13 FIPR-12 NGYYGGAYGYGEYGYYGKREAGFGPTQQTK-- 74

F57A8.8 FIPR-13 YG--GYEYGSPY-GYVGKRSSGFGPKN----- 78

F22B7.4 FIP-1 YGNGGYG-YGNGGGYYGGYN-GY--------- 63

T27E4.4 FIP-2 YGYGGYGGYGGYGG-YGGYG-GYGYKK----- 89

. * * *:

F13E9.2 FIPR-14 MNFYSLSVFIALVFSFNAADGHRCHRRG---NGEYGGGSEEVVVIGAEKPDNV 50

F13E9.3 FIPR-15 MNFYSLFVFIALIFSFNVVHGHRCHRGG---NGGYGGGSGEVVVIGAEKPKDK 50

C06E1.5 FIP-3 MNVYSVFIFAILAISS--ASGIFLPGGGGKKCGGYGGGYGSGVIIGAERPKK- 50

C06E1.6 FIPR-16 MIVYSVFIFAVLAISS--VTGIFLPGGGGKKCGGHG-EYGSGVIIGAERPKK- 49

Y48A6B.4 FIPR-17 MNVYSVCVFAILAISS--VSGIFLPQGG-KKCGGSS-GYGSGVIIGAPRPQ-- 47

H14A12.7 FIPR-18 MNFYNIFIFAVLSIAA--VSGHRC-RGG---YSGGGRGRGG-IVIGTAKE--- 43

Y69F12A.3 FIPR-19 MNFYNIFIFAVLSIAA--VSGHRC-RGR---YSGGGRGRGG-IVIGTAKE--- 43

H14A12.6 FIPR-20 MNFYNIFIFAVLSIAA--VSGHRC-RGG---NNYGGGGRG--IVIGTAKE--- 42

* .*.: :* * :: . * . . ::**: :

F41E7.4 FIP-4 MNFVTFFLLAVLAVAALAAPQRVIEKTTVIRGGGGGFGGG-------GFG-RPAP-FRPP 51

F41E7.5 FIPR-21 MNSYSIILLLGLLAAVMAAPQRVIEKTTIIRGGGGGFGGGPGQFGRGGFGGGPGSNYGPG 60

** :::** * .*.:***********:*********** *** *.. : *

F41E7.4 FIP-4 PPRFG--------------PSFG-------------RPQTTIVKQTIIRG-- 74

F41E7.5 FIPR-21 RGGFGGNGGFGGNGGFGGGPSYGGRGGFGGGPGFGGRGGPTIIKETIIRGRK 112

** **:* * .**:*:*****

C37A5.2 FIPR-22 MKFISVFLVAILAIGAFCATETESKMKMGGSGSDVKTQMNMNSQAPHTLERRSEAQSSMK 60

C37A5.4 FIPR-23 MKFISVFLVAILAIGAFCATETESKMKMGGSGSDVKTEMNMNSQTPHTLERRSETQSSMK 60

C37A5.8 FIPR-24 MKFVSLFLLAILAIGALSDTVTTTKMKM-------------------------------- 28

***:*:**:*******:. * * :****

C37A5.2 FIPR-22 LDGQKSGVKTEMSMDSQTPQIRDTRSETETKMKMEGQGSDMKTEINQQMDTPHTLERRSE 120

C37A5.4 FIPR-23 MDGQGSGVKTEMNMNSQTPQIRDTRSETETKMKMEGQGSDMKTEINQQMDTPHTLERRSE 120

C37A5.8 FIPR-24 ---------------------------------------EMK------------------ 31

:**

C37A5.2 FIPR-22 TESQIKMEGQGSGVKTEMNQQMDAEAPHIRDRRWGYYGGMGGYGMGYGMGYGGMGYGMGM 180

C37A5.4 FIPR-23 TESQIKMEGQGSGVKTEMNQQMDAEAPHIRDRRWGYYGGMGGYGMGYGMGYGGMGYGMGM 180

C37A5.8 FIPR-24 ---PVAMPAQH---QTEMHSEMDSEAPHTRNRRWGYYGGYGGYGR---MGYG----GYGM 78

: * .* :***:.:**:**** *:******** **** **** * **

C37A5.2 FIPR-22 RRFGYGYPMWG 191

C37A5.4 FIPR-23 RRFGYGYPMWG 191

C37A5.8 FIPR-24 RGYGYGYPMWG 89

* :********

F53B6.9 FIP-5 MANQQRLFVFFVVLLFAIVTYAHHSHSYSHSHEHHHHHHGGYYGGGYGGYQPYYGGGGYN 60

T06A1.7 FIPR-25 MANQQKLLLLFVVLLIAIVTYAHHHD--SDSHEHHHHH---------------------- 36

F53B6.8 FIPR-26 ----MDFGILLVFLMAVAGTFAGISVSFSHSHEFPRHG--------------LLGGGGFN 42

: :::*.*: . *:* *.***. :*

F53B6.9 FIP-5 GYNGYYRQNYWGRKK 75

T06A1.7 FIPR-25 ---GMVSE------- 41

F53B6.8 FIPR-26 PYGGYYQSNYWGKRK 57

*

Y51H4A.10 FIP-6 ------------------MKVFLLLLICLATMAYSQPDENNNGFIQRN--FFP------C 34

Y51H4A.32 FIPR-27 MASSSVFTIHSFSASQQKMKVFLLLLICLATLTFSQPDENNNGFIQRN--FFP------C 52

Y51H4A.26 FIPR-28 ------------------MKVFLLLL--LVTLAYSQPDENNNGFIQNDDEYLPADLAKKC 40

H06I04.7 FIPR-29 ---------------MPNSSILLIFL--LTIVILIAPEENHKTYCEVLTSNNS-----NC 38

.::*::* *. : *:**:: : : . *

Y51H4A.10 FIP-6 TVY--CPDNQLCVHGQCVD----------------------------PKEEPP--RFTE- 61

Y51H4A.32 FIPR-27 TVY--CPDNQLCVHGQCVD----------------------------PKEEPP--RFTE- 79

Y51H4A.26 FIPR-28 ENYFDCQQYQTCIFGHCVRKCTSERDCPLGYICFKNGDGQRCIKKNCPKRCPYGCKYGEC 100

H06I04.7 FIPR-29 TAY--CKEKPECSWGRCEG-------------------------YFKTNCVCYSDKYQFC 71

* * : * *:* .: ::

Y51H4A.10 FIP-6 -------

Y51H4A.32 FIPR-27 -------

Y51H4A.26 FIPR-28 VPRPDSQ 107

H06I04.7 FIPR-29 E------ 72

F41E6.10 FIP-7 MHPPYTNRSFFVCCYDVYLHFNIHFTLFLSHKKLPYEKTGREPPLFSLVVGEDVD

EDVGKSKNDDVDGEEQVEEEGSDIFQTLSIKNKLNFVNCG 95
