# Supplementary material for: Anti-Fungal Innate Immunity in C. elegans Is Enhanced by Evolutionary Diversification of Antimicrobial Peptides
Source: PLoS Pathog. 2008 Jul 18;4(7):e1000105. doi: 10.1371/journal.ppat.1000105 (PMC2453101; doi:10.1371/journal.ppat.1000105)
Supplement: Figure S6 — Alignment of the promoter sequences of the genes from the nlp-29 locus. Raw output from CLUSTALW multiple alignments for the proximal 500 bp 5′ sequences for the 6 genes of the nlp-29 cluster. Putative minimal GATA sites are highlighted in yellow; one is shared between 3 nlp genes. (0.06 MB DOC) [file ppat.1000105.s007.doc]

Figure S5

Clustal w (1.83) multiple sequence alignment

*nlp-29* ---ATATTATGATTGCGCGGGAAATCAAATTTTATTACCCATTTAAAAAATTTTGCAAAA 57

*nlp-30* -----CTTAAAAATTTCCTTGAAATGAAATTGTA--AACAACTTTTAAAGTTCAAAAATA 53

*nlp-28* TTGGAAAAAGAGATTTTCTTGAGTTGGCACAACACGGTAAACTTTGAAATTTGCCGCTCA 60

*nlp-31* -----------TTCCCCTCCATATTTATACGTCCTCATCTAACTT---GTTCACCGAATA 46

*nlp-27* ----------AAATTTACTCTAGCTTCATTGCTCCCATTTTGACT--GACTTTGAGACCG 48

*nlp-34* -------------AGAAAGTGGATTCCAAAAACACCAAACACTTTT--ACTTTCTGAATT 45

* *

*nlp-29* CTGAAAAAAATCTTTTCAAA--AATTTAT--AGAAATTCTCTAATTTTTTAAATAGTTTT 113

*nlp-30* TCAATCAATGATATTTTATG--GTTTCA---AATAACTAAAAAAACCACGAAAGCGATGA 108

*nlp-28* CCCCTGTTCGGTATATTGAGTCATTTTGGGCAAAAACCCCACAAACGCTACTCCACTCTC 120

*nlp-31* ATCGTCCTCATTCGTTTCCCCGTCTTCTTCTAGTTTCTACCTCGTTTTCTCTGATCTATG 106

*nlp-27* TTTTTGAAAAACTTCTCGAA--ACTCTTAATGTAAAGTAAATTTATTATCAAAATTTATA 106

*nlp-34* TATTTTAGAAAATATTTAACTTGCTCTAA--ACTTTCTAATTTTTATTTTGACAGTTTCG 103

* *

*nlp-29* ATTTGGTCGTTACGGTCAGTT-TTCTCTCATTTGA-AGATAAAATCGCCTCTATC---CC 168

*nlp-30* GCGCACTTTCCA-GATCAAAT-ATATTTCATTTCCTAGAAAAAAGCTGTGTTTTT---CT 163

*nlp-28* AAATATGTTTGAAAATTGACTGAGATGTTGGTTTA-ATTTTGAAGCTGCCTAAAAAG-TT 178

*nlp-31* ACGC-TTTCTGATCTTTAATTGTCGACTCCACTCT--ATCAGTCCCAGTTCTTCGTG-TC 162

*nlp-27* ATTGTTTCTCTATATTTTCAT-AGAACTTTCCTCAAAAAAAACTACGACGCTACTTTTTC 165

*nlp-34* GATCTCTCAAAGACTTTGTCTACAAACATCTCTTAAAAATTCAAAAAATGCATTG---TT 160

* * *

*nlp-29* AGCTTAAAGTATAACAAAAATGCGGTTTTGCCACGCTTTAGGCAG--TAAAAAAAACGGT 226

*nlp-30* AAATCGTTCGAAAGTCCAAATTATGTCAAGCACGGACTGTGATAGCTTCAAAAGTACAAT 223

*nlp-28* AAAATCCACTATTATAAAAGTACAAA-ATGCTTAAAATCTTTCATATTATGTAACAAAAA 237

*nlp-31* ACCACCATCGTTACCAAATGTGTTATTCGGCGATCGGCAAGTGAGAGAAGCCGATTTCCC 222

*nlp-27* ACAGTAACTTTTTATACAAATGTATTTGGCAACAAAAAAACATTTCTGATTTGTCATTTT 225

*nlp-34* AGTCTTATTTTACATATACATACCTATTATACCTGACATACCTAG---ACCTATTCCTAC 217

* * *

*nlp-29* T--TGTGTATGTGTTCAGAATGTAGGTGATA-TACATATATTTTTTGACCGTTTTCAATT 283

*nlp-30* CACTGATTATTTGATTATAAATTCAGTGAT----CA-ATACTTTTTAAACGCTTCAGTTT 278

*nlp-28* TCATATTT-CGTATCAAACACAAATACCACG-TTCCAATATTTTATAACGGCTAACGTTA 295

*nlp-31* CGAAAGTTTTGTGATTTTGCCCGGCAATGGA-TTAGTCTGTTTGGTGAACAGGGATGAAA 281

*nlp-27* CCATGGAT-CATCTTGACAAACATCAAAACACTTGAGAAAAATTGCGTATTTTGCCCGAT 284

*nlp-34* CTAGACCTAGACCTACCTAAACCTAGACCTA--CCTAGACCTAGACGTACCTAGACGTAG 275

*

*nlp-29* TCCTTGT--TGATTTGGAATTGATCTGTAGGTGAT--TAAGCATCCAACTCTATTTG--A 337

*nlp-30* TTATTACAATTGGTTGGAATTGATCTATATTTTAT--TAAGCTTTCAAATCTGTTCG--T 334

*nlp-28* CCACTGT--CCAATTTGAACTGATCTATAACTGAT--CTAGAATCAAACTTTATTTG--A 349

*nlp-31* CCGTGTAGACCATCACACAGTGGT---TGATCTTC--TTCGTGACCGCGTCTGATAA--G 334

*nlp-27* AGGCCATTACCAATTTGACCAAGTGACCAATTCAAAGTGATTCACCAAGTCAGTTCGCCG 344

*nlp-34* ACCATACACCAATTTTTCATTGGTAGACATTTTTCAATTCACATGTGCCCTTGATGA--- 332

* *

*nlp-29* CGTACAAGGAGAGATCTTAGATGAAAAAGAAACAGAGTCTCGTGATGACATCATCGGTTG 397

*nlp-30* CGTATAAGGAGAGATATCAGAGGGAAAAGAAACAGAGTCTTGTGATGATATCATTTTTTG 394

*nlp-28* CGTATAAC-AGTGATGTTCAA--GTAAAAATACCGAGTCTTGTGAT----------CTTG 396

*nlp-31* ATAGTGTGTTGGAGTGAGAGAGGGAGAAGAAGGAAAATGGTTTAATGATGTCATT-GTTT 393

*nlp-27* CGTGCAGGAGGTGGAGATGTGGTAGAAAAAAAAAAGATGATCTTATCA----GTTAGTTG 400

*nlp-34* TTTTTGACCTACTAGATAGTACTATCCATGTTGCGAATTACGTCACCAAAG-GTCAATAC 391

* * * * *

*nlp-29* ATCGGCGAAAAGGA--AATTGAAGATACGGGAAACCTTTCTATAAAAGAGGC-GAAAAAC 454

*nlp-30* ATCGCCAAAGAGAA--AATTGAAGATAGTGGAAATCTTG-TATAAAAGTCAC-AAAAGCT 450

*nlp-28* ATCAACGAAAAAAACTAATTGAAGATAGACGACGGCACC-TATAAAAGAGGC-GAAAAAC 454

*nlp-31* CTCGCCAAAAAGAGA-TAGTGGAGATAAGAATATCTGATCTATAAAA-AGAC-AAGTAAA 450

*nlp-27* TTTGACGAAAAAGG--AACCCAAAATGAACAG--------TATAAAAGATGCCAGGTAAC 450

*nlp-34* ATACATCTCTAAAATTTTCTGACAGTTTCTCATGATTATCTCAAGGGGTCTCCCTATTTT 451

* * * * * *

*nlp-29* GTGGTAATAATCAGAAAG-TCATTCACTTGATTTTTAAAA---AACAAAA- 500

*nlp-30* GTGGAAAAAATCAGAAAG-CCATTTGCTTGATTTTTAAAATAAAACAAAAA 500

*nlp-28* GTGGTAAACATCAGAAAA-TCATTTTCGTGACTTTTAAAAT---ACAAAA- 500

*nlp-31* GTGGCGAATATTAGAAAACTCATATTCTTGATATTCTACAATCAATAACA- 500

*nlp-27* CAGGTAGAATACAGAAAACTTATTTACAAGTTTTTCAAAGAAATATCATT- 500

*nlp-34* TACACGATTAACAAAAAT-TGGGATGCCATAGTTTTCAATTTCCAGAATC- 500

* *** * ** * * *
