# Supplementary material for: Anti-Fungal Innate Immunity in C. elegans Is Enhanced by Evolutionary Diversification of Antimicrobial Peptides
Source: PLoS Pathog. 2008 Jul 18;4(7):e1000105. doi: 10.1371/journal.ppat.1000105 (PMC2453101; doi:10.1371/journal.ppat.1000105)
Supplement: Table S2 — Results of the analysis of adaptive sequence evolution for individual branches of the nlp tree. (0.06 MB DOC) [file ppat.1000105.s009.doc]

**Table S2**

Results of the analysis of adaptive sequence evolution for individual branches of the *nlp* tree.

| Branch^a^ | b ratios^b^ |  |  | 2 ratios^b^ |  |  |
| --- | --- | --- | --- | --- | --- | --- |
|  | *d_N_*/*d_S_* | bootstrap |  | *d_N_*/*d_S_* | 2Δ*L* | *P* |
| A | <0.001 | 98 |  | <0.001 | 1.052 | 0.3050 |
| B | **1.264** | 65 |  | **>999** | 4.401 | 0.0359 |
| C | **41.038** | 61 |  | **11.722** | 5.173 | 0.0229 |
| D | 0.254 | 71 |  | 0.110 | 0.157 | 0.6919 |
| E | <0.001 | 100 |  | <0.001 | 1.176 | 0.2782 |
| F | <0.001 | 81 |  | <0.001 | 0.026 | 0.8729 |
| G | 21.790 | 44 |  | 0.091 | 0.027 | 0.8705 |
| H | 0.883 | 50 |  | 14.681 | 0.196 | 0.6579 |
| I | 2.699 | 49 |  | 0.002 | 0.104 | 0.7472 |
| J | <0.001 | 98 |  | 0.001 | 2.546 | 0.1106 |
| K | **117.409** | 73 |  | >999 | 0.078 | 0.7794 |
| L | 0.004 | 70 |  | 0.024 | 0.456 | 0.4995 |
| M | <0.001 | 88 |  | >999 | 0.680 | 0.4096 |
| N | <0.001 | 96 |  | <0.001 | 0.841 | 0.3591 |
| O | <0.001 | 99 |  | <0.001 | 1.420 | 0.2334 |
| P | <0.001 | 97 |  | <0.001 | 1.014 | 0.3140 |
| Q | <0.001 | 78 |  | <0.001 | 0.000 | 0.9980 |
| R | <0.001 | 77 |  | <0.001 | 0.003 | 0.9584 |
| S | **88.990** | 52 |  | **>999** | 6.368 | 0.0116 |
| T | 0.373 | 70 |  | **2.194** | 4.057 | 0.0440 |
| U | 0.040 | 61 |  | 0.036 | 0.384 | 0.5357 |

a, branch as depicted in Fig. S2B.

b, *d_N_*/*d_S_* rate ratios for individual branches were inferred with the b ratio model, in which all branches were allowed to vary; the optimal model had a likelihood of ln *L* = -791.49; the significance of individual branches having a *d_N_*/*d_S_* rate ratios above 1 or below 1 was assessed with non-parametric bootstrapping using 100 replicates; *d_N_*/*d_S_* rate ratios larger than 1 and with bootstrap support of more than 50 are given in bold.

c, *d_N_*/*d_S_* rate ratios were inferred with the 2 ratio model, in which only the branch of interest was allowed to differ from the remaining branches; the significance of the individual branches to be different from the remaining branches was assessed via a likelihood ratio test comparison to the null model, in which all branches of the tree were assumed to have identical *d_N_*/*d_S_* rate ratios; the null model had a likelihood score of ln *L* = -814.40; the probability *P* was calculated from twice the likelihood difference 2Δ*L* between null model and tested model; bold *d_N_*/*d_S_* rate ratios indicate those that are larger than 1 and supported by a nominal *P* value smaller than 0.05.
